# The association of center volume with transplant outcomes in selected high-risk groups in kidney transplantation

**DOI:** 10.1186/s12882-023-03099-0

**Published:** 2023-03-20

**Authors:** Massini Merzkani, Su-Hsin Chang, Haris Murad, Krista L. Lentine, Munis Mattu, Mei Wang, Vangie Hu, Bolin Wang, Yazen Al-Hosni, Obadah Alzahabi, Omar Alomar, Jason Wellen, Tarek Alhamad

**Affiliations:** 1 Division of Nephrology, Washington University School of Medicine, 4523 Clayton Ave. CB 8126, St. Louis, MO 63110 USA; 2 Division of Public Health Sciences, Department of Surgery, Washington University School of Medicine, St. Louis, MO USA; 3 Center for Abdominal Transplantation, Saint Louis University, St. Louis, MO USA; 4 Brown School of Social Work, Washington University in St. Louis, St. Louis, MO USA; 5 Department of Surgery, Washington University in St. Louis, St. Louis, MO USA; 6 Transplant Epidemiology Research Collaboration (TERC), Institute of Public Health, Washington University School of Medicine, St. Louis, MO USA

**Keywords:** Transplant center volume, Patient survival, Graft failure, Graft loss, Kidney allograft failure

## Abstract

**Background:**

In context of increasing complexity and risk of deceased kidney donors and transplant recipients, the impact of center volume (CV) on the outcomes of high-risk kidney transplants(KT) has not been well determined.

**Methods:**

We examined the association of CV and outcomes among 285 U.S. transplant centers from 2000–2016. High-risk KT were defined as recipient age ≥ 70 years, body mass index (BMI) ≥ 35 kg/m^2^, receiving kidneys from donors with kidney donor profile index(KDPI) ≥ 85%, acute kidney injury(AKI), hepatitisC + . Average annual CV for the specific-high-risk KT categorized in tertiles. Death-Censored-Graft-Loss(DCGL) and death at 3 months, 1, 5, and 10 years were compared between CV tertiles using Cox-regression models.

**Results:**

Two hundred fifty thousand five hundred seventy-four KT were analyzed. Compared to high CV, recipients with BMI ≥ 35 kg/m^2^ had higher risk of DCGL in low CV(aHR = 1.11,95%CI = 1.03–1.19) at 10 years; recipients with age ≥ 70 years had higher risk of death in low CV(aHR = 1.07,95%CI = 1.01–14) at 10 years. There was no difference of DCGL or death in low CV for donors with KDPI ≥ 85%, hepatitisC + , or AKI.

**Conclusions:**

Recipients of high-risk KT with BMI ≥ 35 kg/m^2^ have higher risk of DCGL and recipients age ≥ 70 years have higher risk of death in low CV, compared to high CV. Future studies should identify care practices associated with CV that support optimal outcomes after KT.

**Supplementary Information:**

The online version contains supplementary material available at 10.1186/s12882-023-03099-0.

## Introduction

Kidney transplantation (KT) is the treatment of choice for end stage renal disease (ESRD), as it improves quality of life and reduces the mortality rate of patients with ESRD, compared to dialysis, at lowest costs to the healthcare system [1]. The growth in the number of patients on the waiting list far exceeds the rate at which kidney transplantation is performed [2, 3]. To narrow this gap, several strategies have been used to expand the pool of deceased donors. Importantly, even high-risk KT is cost-effective compared to dialysis [1].

Strategies to increase deceased donor pool include the use of high-risk deceased donors, e.g., kidneys from donors with high kidney donor profile index (KDPI), acute kidney injury (AKI), or hepatitis C positivity. For donors with AKI, it has been shown that recipients had similar graft survival at both short- and long-term compared to recipients of kidneys from donors without AKI [4, 5]. Recent data have shown that patients receiving kidneys from donors with Hepatitis C viremic did not experience increased risk for graft loss compared to those receiving kidneys with no viremic donors [6]. KDPI ≥ 85% is associated with lower graft survival [7], but is imprecise and carries chance of misclassifying risk, which may increase the likelihood of organ discard.

In this context, the complexity of recipients is increased with the growing number of elderly and obese candidates on the waiting list [8]. Older recipient age has been associated with increased comorbidities, frailty, and risk of infection and death with functional allograft, when compared to younger population [9, 10]. Obesity has been also associated with an increased risk for delayed graft function, proteinuria, rejection, and graft failure in transplant recipients [11, 12].

Prior studies in solid organ transplants have shown an association between transplant center volume (CV) and patient outcomes. Centers performing relatively fewer solid organ transplants may have inferior allograft outcomes, whereas high-volume centers are associated with improved survival outcomes [13–21]. However, there are no large-scale studies that examined this relationship on high-risk KT, defined as recipient age ≥ 70 years, body mass index (BMI) ≥ 35 kg/m^2^, or receiving kidneys from donors with KDPI ≥ 85%, AKI, or hepatitis C antibody positivity. This is particularly important in the modern era with the growing number of high-risk donors in the donor pool and high-risk patients in the waitlist candidates.

## Methods and materials

### Study population

**Fig. 1:**
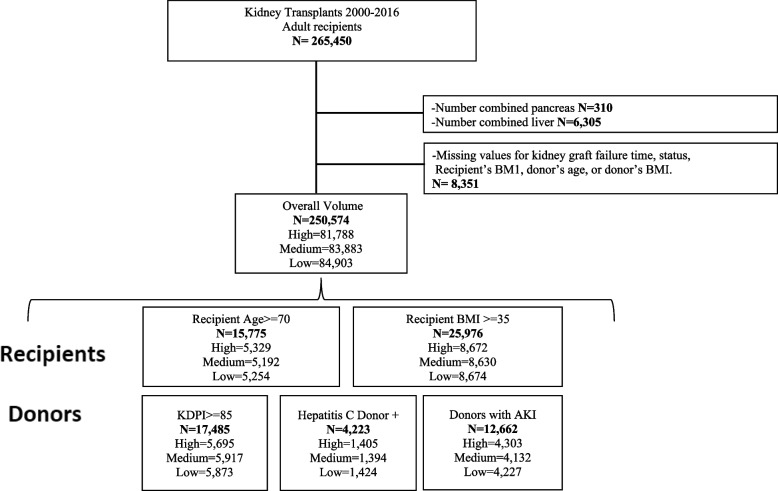
Flowchart

The study cohort was composed of all adult patients age ≥ 18 years, who received a solitary KT between 2000 and 2016. Data from the Organ Procurement and Transplantation Network (OPTN) were used. This study was exempt of IRB and no informed consent was needed, as this is a data registry. Exclusion criteria were combined kidney-liver and kidney-pancreas (*n* = 6,615), transplants with missing values for kidney graft failure time or status, recipient's BMI, donor’s age and donor's BMI (*n* = 8,351), resulting in 250,574 kidney transplants (Fig. 1).

This study cohort was then divided into the following high-risk kidney transplant groups; a) recipient age at the time of transplant ≥ 70 years old (*n* = 15,775); b) BMI at transplant ≥ 35 kg/m^2^ (*n* = 25,976); patients receiving kidneys from donors with: c) KDPI ≥ 85% (*n* = 17,485); d) AKI (serum creatinine ≥ 2 mg/dl) (*n* = 12,662); and e) or hepatitis C antibody positive (HCV +) (*n* = 4,223).

### Transplant center volume

For the overall population and for each of the high-risk KT groups, transplant center volume was categorized into tertiles: low-, medium-, and high-volume based on their average annual volume of that KT. Therefore, the tertiles cutoffs were different across these high-risk KT groups. For each of the high-risk group were also divided in to tertiles to determine the volumes for this specific characteristic as shown in Tables 1 and 2.

**Table 1:** Baseline characteristics for the cohort and individual high-risk group

**Variables**	**Entire group (***N* = 250,574)				**Age older than 70 years old** (*N* = 15,775)				**Recipient BMI > 35 kg/m2 (***N* = 25,976)			
	**Low (*n* = 84,903)**	**Medium (*n* = 83,883)**	**High (*n* = 81,788)**	***p*-value**	**Low (*n* = 5254)**	**Medium (*n* = 5192)**	**High (*n* = 5329)**	***p*-value**	**Low (*n* = 8674)**	**Medium (*n* = 8630)**	**High (*n* = 8672)**	***p*-value**
**Recipient and Transplant Factors**												
Age (%)				< .0001				-				0.4027
18–35 years	16.9%	16.7%	16.1%		-	-	-		13.3%	12.4%	12.9%	
36–50 years	30.4%	31.1%	30.5%						34.1%	34.7%	34.4%	
51–65 years	39.0%	39.3%	39.3%		-	-	-		42.9%	42.9%	42.3%	
> 65 years	13.7%	12.9%	14.1%		100%	100%	100%		9.7%	10%	10.4%	
Gender (%)				< .001				0.9744				0.0078
Male	61.5%	60.1%	60.0%		66.2%	66.4%	66.2%		55.3%	52.9%	54.3%	
Female	38.5%	39.9%	40.0%		33.8%	33.6%	33.8%		44.7%	47.1%	45.7%	
Race (%)				< .0001				< .0001				< .0001
White	55.5%	48.5%	54.8%		66.1%	67.4%	68.1%		54.2%	49.1%	54.7%	
Black	25.1%	28.1%	23.3%		18.2%	16.6%	14.9%		28.8%	38%	31.4%	
Hispanic	12.9%	16.0%	13.8%		9.8%	9.7%	8.8%		12.7%	9.5%	11.1%	
Other	6.4%	7.4%	8.1%		6%	6.3%	8.3%		4.3%	3.4%	2.8%	
BMI (kg/m2)	27.8	27.5	27.6	< .0001	27.5	27.3	27.4	0.1098	37.71	37.91	38.22	< .0001
Time on dialysis (months)				< .0001				< .0001				< .0001
0	20.0%	21.1%	26.8%		20.6%	22.5%	28.1%		16.5%	17.1%	22.6%	
1–12	9.6%	8.7%	10.2%		7%	7.9%	7.3%		7.9%	7%	9%	
> 12 to 24	14.7%	13.9%	13.3%		15.2%	15.1%	14.7%		14.2%	12.5%	14.3%	
> 24	55.6%	56.4%	49.7%		57.3%	54.5%	49.9%		61.4%	63.4%	54.1%	
Time on waiting list (years)				< .0001				0.0048				< .0001
0–1	41.7%	41.0%	43.3%		37.8%	40%	40.3%		40.4%	36%	43.4%	
1 to 3	35.2%	35.0%	32.2%		36.9%	37.9%	37.3%		36.2%	35.9%	32.5%	
> 3	23.1%	24.0%	24.5%		25.3%	22.1%	22.4%		23.4%	28.1%	24.1%	
HLA Mismatch Level				0.0006				0.2117				< .0001
0	9.2%	9.1%	9.2%		6.4%	7%	6.2%		8.7%	8.9%	8.8%	
1 to 2	12.2%	12.0%	12.5%		12.1%	11.4%	11.1%		10.9%	10%	12.4%	
3 to 6	78.5%	78.9%	78.3%		81.6%	81.6%	82.8%		80.4%	81.1%	78.8%	
PRA (%)				< .0001				< .0001				< .0001
0	57.8%	56.8%	59.4%		63.1%	67.8%	66.2%		58.7%	56.9%	60.9%	
0–20	18.1%	16.0%	15.5%		17%	14%	15.7%		16.8%	15.9%	15%	
20–80	13.8%	15.2%	14.3%		13.4%	11.4%	11.9%		14.5%	15.4%	14.2%	
> 80	10.2%	12.0%	10.8%		6.5%	6.8%	6.2%		9.9%	11.8%	9.8%	
Induction				< .0001				< .0001				< .0001
Basiliximab	29.2%	22.3%	20.2%		33.2%	34.3%	25.9%		23.5%	23.7%	14.1%	
Alemtuzumab	7.7%	11.0%	13.8%		7.9%	8.3%	9.5%		10.2%	8.9%	21.7%	
Thymoglobulin	41.1%	44.0%	47.4%		37.1%	38%	47.1%		47.1%	45.7%	45.8%	
Other	0.7%	0.5%	0.9%		0.5%	0.2%	0.4%		0.3%	0.6%	0.8%	
Missing	21.3%	22.2%	17.8%		21.3%	19.1%	17.1%		18.8%	21.1%	17.6%	
Year of transplantation				< .0001				< .0001				< .0001
2000 ~ 2005	34.1%	30.8%	28.2%		22.8%	23.6%	19.8%		28.1%	25.3%	23.5%	
2006 ~ 2011	36.0%	37.0%	37.1%		38%	40.4%	40.2%		37%	39.7%	38.8%	
2012 ~ 2016	29.9%	32.2%	34.7%		39.2%	36%	40%		34.9%	34.9%	37.7%	
**Donor Factors**												
Age (years)	38.9	38.2	39.9	< .0001	45.6	47	47.5	< .0001	39.38	39.64	40.19	0.0024
Gender				< .0001				0.02				< .0001
Male	53.1%	53.3%	51.2%		52%	51.5%	49.4%		54.1%	55.2%	51.5%	
Female	46.9%	46.7%	48.8%		48%	48.5%	50.6%		45.9%	44.8%	48.5%	
Race				< .0001				< .0001				< .0001
White	72.6%	67.2%	69.8%		75.4%	76.5%	73.1%		73.7%	70.3%	71.7%	
Black	11.9%	13.8%	12.2%		10.9%	10.5%	10.6%		11.6%	16.8%	14.9%	
Hispanic	12.0%	14.4%	13.5%		9.8%	9.7%	11.4%		11.2%	9.9%	11.1%	
Other	3.6%	4.5%	4.4%		3.9%	3.3%	4.9%		3.6%	3%	2.3%	
BMI	26.9	27.0	26.99	0.001	27.4	27.8	27.5	0.02	27.64	27.78	28.11	< .0001
Hypertension	17.4%	18.3%	16.7%	< .0001	30.8%	32.4%	32.7%	0.09	20.8%	21.6%	18.9%	< .0001
Cause of death				< .0001				< .0001				0.35
Anoxia	22.4%	23.6%	25.1%		22.4%	23.7%	24.5%		24%	25.5%	25.2%	
CVA	34.4%	35.0%	35.2%		43.8%	46.9%	47.6%		34.2%	34.2%	34.1%	
Other	43.2%	41.4%	39.6%		33.8%	29.4%	28%		41.7%	40.3%	40.7%	
Cold ischemia time (hours)				< .0001				< .0001				< .0001
< 12	54.6%	52.6%	58.4%		48%	46%	47.2%		53.1%	50.5%	58.2%	
12 to 24	34.6%	34.8%	26.7%		40.1%	41.2%	28.8%		36%	36.1%	27.6%	
> 24	10.9%	12.6%	15.0%		12%	12.9%	24%		10.9%	13.4%	14.2%

**Table 2:** Baseline characteristics for the cohort and individual high-risk group

**Variables**	**KDPI ≥ 85% (*N* = 17,485)**				**Donor with AKI (***N* = 12,662)				**Donor with Hepatitis C + (** *N* = 4,223)			
	**Low (*n* = 5873)**	**Medium (*n* = 5917)**	**High (*n* = 5695)**	***p*-value**	**Low (*n* = 4227)**	**Medium (*n* = 4132)**	**High (*n* = 4303)**	***p*-value**	**Low (*n* = 1424)**	**Medium (*n* = 1394)**	**High (*n* = 1405)**	***p*-value**
**Recipient and Transplant Factors**												
Age (%)				< .0001				< .0001				0.16
18–35	4.0	3.0	3.5		11.8%	11.9%	10.2%		2.8%	1.8%	1.9%	
36–50	14.5%	15.6	14.5%		29.2%	30.1%	27.1%		25.1%	24%	24.5%	
51–65	48.4%	51.5%	48.7%		43.2%	41.4%	42.5%		60.5%	61.5%	63.4%	
> 65	33%	30%	33.3%		15.8%	16.7%	20.1%		11.5%	12.7%	10.2%	
Gender (%)				0.67				0.19				0.11
Male	62.2%	62.9%	62.2%		62.4%	60.9%	62.7%		80.8%	82.5%	79.4%	
Female	37.8%	37.1%	37.8%		37.6%	39.1%	37.3%		19.2%	17.5%	20.6%	
Race (%)				< .0001				< .0001				< .0001
White	50.8%	38.3%	37.3%		45.2%	44.5%	30.7%		28.9%	21.7%	18.4%	
Black	28%	44.3%	33.5%		31.7%	41.5%	28.4%		55.8%	68.7%	71%	
Hispanic	12.5%	10.7%	16.5%		15.5%	8.8%	27.7%		11.8%	7.1%	7.5%	
Other	8.7%	6.6%	12.7%		7.6%	5.2%	13.2%		3.6%	2.4%	3.1%	
BMI (kg/m^2^)	28	28	27.2	< .0001	28.2	28.3	27.7	< .0001	27.23	27.24	26.92	0.053
Time on kidney waiting list				< .0001				< .0001				0.003
0–1 year	31.1%	26.8%	30.7%		30.3%	27.7%	26.3%		57.9%	59%	64%	
1 to 3 years	40.5%	41%	39.4%		38.2%	36.7%	38.3%		32%	30.8%	28.7%	
> 3 years	28.4%	32.2%	29.9%		31.5%	35.6%	35.4%		10.2%	10.2%	7.3%	
HLA Mismatch Level (%)				0.002				< .0001				0.1068
0	4.3%	3.7%	3.2%		6.5%	6.1%	3.7%		1%	0.7%	0.5%	
1 to 2	4.9%	4.1%	4%		6.2%	6.5%	5.3%		3.6%	3.4%	2.2%	
3 to 6	90.8%	92.2%	92.8%		87.2%	87.5%	91%		95.4%	95.9%	97.3%	
PRA (%)				< .0001				< .0001				< .0001
0	60.1%	61.9%	65.9%		56.2%	55.2%	63.5%		69.1%	60.4%	72.7%	
0–20	21.9%	18.2%	17.5%		18.7%	16.9%	14.1%		15.2%	21.2%	12.3%	
20–80	13.1%	14.6%	11.9%		14.1%	14.9%	13.1%		11.5%	14.3%	10.9%	
> 80	5%	5.3%	4.7%		11%	12.9%	9.3%		4.2%	4.1%	4%	
Induction (%)				< .0001				< .0001				< .0001
Basiliximab	23.9%	21.2%	16%		19.1%	18.7%	13.2%		28.4%	26.2%	16.9%	
Alemtuzumab	8.3%	13.3%	14.5%		10.8%	13.8%	10.6%		5.3%	10.8%	2.9%	
Thymoglobulin	49.3%	42.4%	47.7%		51.6%	50.1%	59.5%		44.6%	43.2%	41.9%	
Other	0.5%	0.5%	0.4%		0.4%	0.6%	0.4%		0.8%	0.5%	0.4%	
Missing	18%	22.6%	21.3%		18.1%	16.9%	16.3%		20.9%	19.3%	37.9%	
Year of Transplantation (%)				< .0001				< .0001				0.13
2000 ~ 2005	30.6%	28.2%	27.1%	< .0001	23.6%	17%	17.7%	< .0001	31%	29.6%	30.7%	0.13
2006 ~ 2011	41.2%	42.4%	40.9%		35%	43.1%	33%		31.5%	34.8%	35.5%	
2012 ~ 2016	28.2%	29.4%	32%		41.4%	40%	49.3%		37.4%	35.7%	33.7%	
**Donor Categories**												
Age	59.3	58.7	57.4	< .0001	35.3	35.5	36.9	< .0001	38.97	39.23	40.04	0.02
Gender				< .0001				0.03				0.14
Male	42.7%	43.5%	47.4%	< .0001 < .0001	73.4%	71.3%	71%	0.03 < .0001	66.3%	65.4%	62.8%	0.14 0.005
Female	57.3%	56.5%	52.6%		26.6%	28.7%	29%		33.7%	34.6%	37.2%	
Race												
White	63.7%	53.3%	54.3%	< .0001 0.24	65%	65.7%	55.6%	< .0001 0.002	78.2%	77%	75.4%	0.005 0.97
Black	24.3%	33.1%	27.5%		18.5%	22.9%	17.5%		9.2%	13.3%	12.7%	
Hispanic	8.3%	9.5%	11.7%		13.2%	8.7%	21.7%		11.4%	8.5%	10.8%	
Other	3.7%	4%	6.5%		3.3%	2.6%	5.2%		1.3%	1.2%	1.1%	
BMI	28.1	28.3	28		28.6	28.9	29.2		26.15	26.13	26.32	
Hypertension				< .0001				0.01				0.59
No	27%	24.8%	29.8%	< .0001 < .0001	74.3%	73.4%	71.3%	0.01 < .0001	78%	78.8%	77.2%	0.59 0.22
Yes	73%	75.2%	70.2%		25.7%	26.6%	28.7%		22%	21.2%	22.8%	
Cause of death												
Anoxia	10.8%	11.3%	15.3%	< .0001 < .0001	36.5%	41.6%	46.5%	< .0001 < .0001	29.8%	30.2%	33%	0.22 < .0001
CVA	77.2%	78.7%	72.4%		24.8%	23.4%	26.3%		33.4%	31.7%	32.5%	
Other	12%	10%	12.3%		38.7%	35%	27.2%		36.8%	38.1%	34.5%	
Cold ischemia time (hours)												
< 12	30.2%	26%	18.4%	< .0001 < .0001	25.9%	21.3%	14.9%	< .0001 < .0001	28.9%	22.5%	21.5%	< .0001 < .0001
12 to 24	53.8%	53.1%	42%		56.1%	52.3%	40.3%		52.4%	49.9%	45.6%	
> 24	16%	20.9%	39.5%		18%	26.4%	44.8%		18.7%	27.5%	32.9%	
Total months from diagnosis to transplantation												
0	9.3%	10.5%	12.3%	< .0001	10.3%	12.2%	10.5%	< .0001	8.8%	8.5%	13.1%	< .0001
0–12	5.9%	4.2%	5.7%		4.7%	4.4%	3.3%		10%	7.9%	11.6%	
12 to 24	14%	12%	13.5%		12.3%	11.2%	10.5%		20.9%	20.7%	21%	
> 24	70.8%	73.4%	68.5%		72.7%	72.2%	75.8%		60.4%	62.9%	54.3%	

### Outcomes and covariates

Death Censored Graft Loss (DCGL) was defined as returning to dialysis or receiving another renal transplant. Death was defined as recipient demise. Recipient characteristics included age, gender, race, BMI at time of KT, pre-transplant dialysis, and time on dialysis. Donor characteristics include KDPI, calculated using 10 donor factors including age, height, weight, ethnicity, history of hypertension, history of diabetes, cause of death, serum creatinine, HCV serological status, and Donation after Cardiac Death (DCD) Status, as well as each component separately.

### Statistical methods

Patient characteristics were summarized using proportions for categorical variables and means and standard deviations for continuous variables. Differences between center volume categories (high, medium, low) were compared using χ^2^ test for categorical variables and analysis of variance test or Kruskal Wallis tests for continuous variables, depending on the distribution of the variable. Kaplan–Meier analyses was performed on DCGF and death for the three categories of transplant center volume were compared using Log Rank tests.

Multivariable Cox regression analyses were used to assess the independent association of center volume with the two outcomes (DCGL and death), controlling for all aforementioned recipient and donor characteristics as well as transplant factors e.g., cold ischemic time greater than 24 h, except for the variable used to define high-risk. For each group of the high-risk group were also analyzed with Cox regression for our two outcomes (DCGL and death). DCGL was evaluated at 3 months, 1, 5 and 10 years of follow-up following KT. The results for 10 years are reported in the main text, and the other results are reported in the Supplemental Materials. All tests are two-sided. A *p*-value less than 0.05 was considered statistically significant for all tests. All analyses were performed using SAS 9.4 software (Cary, NC).

## Results

The cohort included 250,574 KT performed in 285 transplant centers between 2000 and 2016 (Fig. 1). Overall, patients transplanted at high volume centers were more likely to be older (age > 65 years), have longer waiting time and cold ischemia time, and to receive T cell depletion (thymoglobulin or alemtuzumab) for induction (Tables 1 and 2). The baseline characteristics of each high-risk group stratified by individual transplant center volume characteristics are described also in Tables 1 and 2.

### Multivariable analysis

#### Death censored graft loss and death for the entire group

**Fig. 2:**
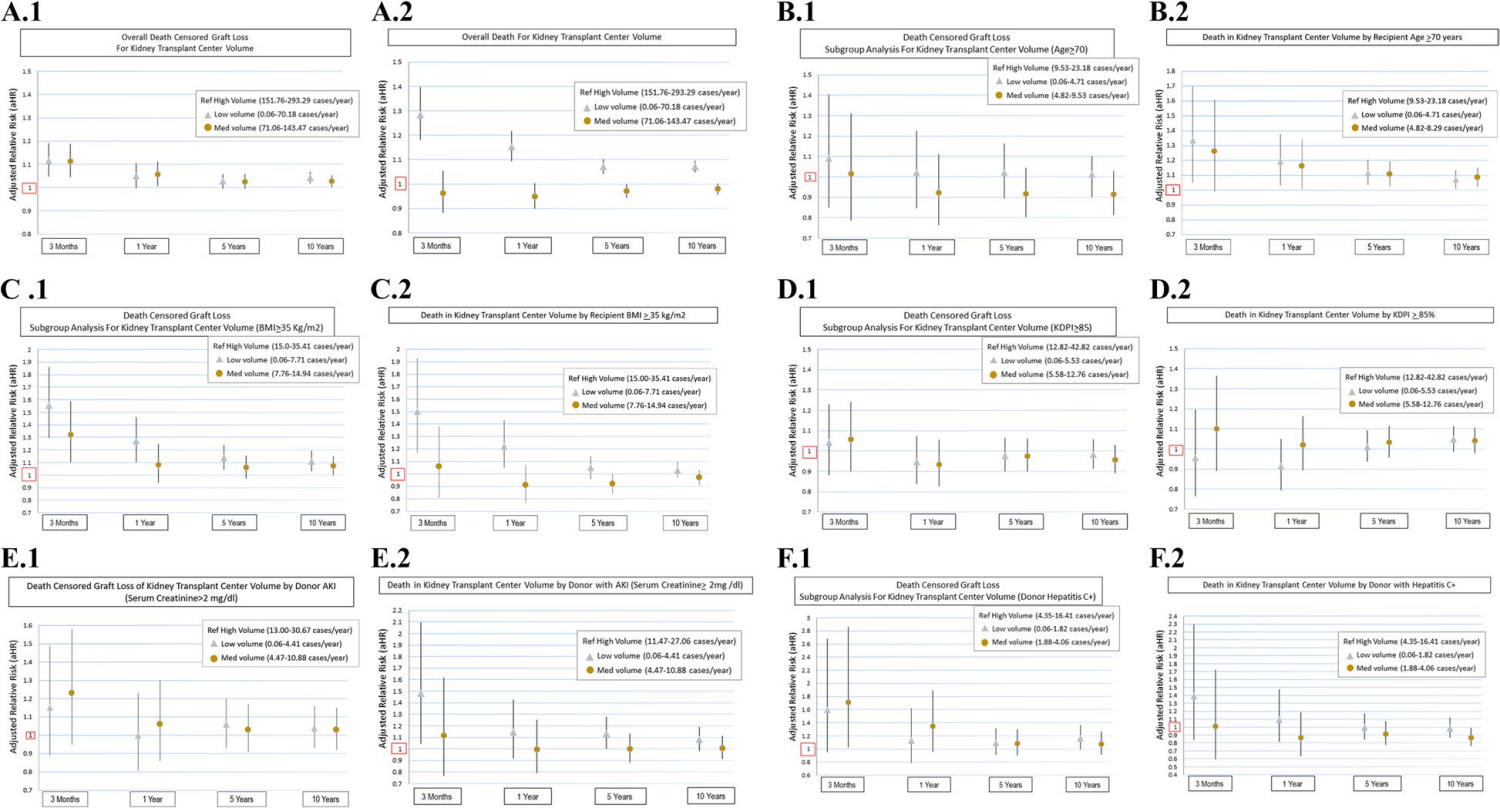
Subgroup Multivariate analysis for transplant center volume. **A.1** Overall kidney transplants center volume associated DCGL. **A.2** Overall kidney transplants center volume associated death. **B.1** Center Volume for Recipient age ≥ 70 years associated DCGL. **B.2** Center Volume for Recipient age ≥ 70 years associated death. **C.1** Center Volume for Recipient BMI ≥ 35 kg/m^2^ associated DCGL. **C.2** Center Volume for Recipient a BMI ≥ 35 kg/m^2^ associated death. **D.1** Center Volume for Transplants with KDPI ≥ 85% associated DCGL. **D.2** Center Volume for Transplants with KDPI ≥ 85% associated death. **E.1** Center Volume for Transplants with Donor AKI with Serum Creatinine ≥ 2 mg/dl associated DCGL. **E.2** Center Volume for Transplants with Donor AKI with Serum Creatinine ≥ 2 mg/dl associated death. **F.1** Center Volume for Transplants with Donor with hepatitis C associated DCGL. **F.2** Center Volume for Transplants with Donor with hepatitis C associated death

Compared with high CV, patients undergoing KT at low CV (adjusted hazard ratio, [aHR] = 1.04; 95% confidence interval [CI], 1.02–1.07) and at medium CV (aHR = 1.03; 95% CI, 1.00–1.05) had higher risk of DCGL at 10 years. Furthermore, low (but not medium) CV volume was associated with higher risk for death at 10 years (aHR = 1.07; 95% CI, 1.05–1.09), when compared to medium center volume (Supplemental Table 1, Fig. 2A.1 and A.2).

#### Death censored graft loss and death for high-risk groups

##### Recipient Age greater or equal to 70 years old

The risk for death at 10 years for recipient age ≥ 70 years, when compared with high CV, was higher in low (aHR = 1.07; 95% CI, 1.01–1.14) and medium CV (aHR = 1.09; 95% CI, 1.03–1.15) **(**Fig. 2B.2). However, no statistically significant difference was observed in risk for DCGL for low, medium CV when compared with high CV (Fig. 2B.1) (Supplemental Table 2).

##### Kidney recipients with Body Mass Index greater or equal than 35 kg/m^2^

The risk for DCGL in patients with BMI ≥ 35 kg/m2 was higher in low volume centers (aHR = 1.11; 95% CI, 1.03–1.19) and for medium CV (aHR = 1.07, 95% CI, 1.00–1.15) when compared with the high CV at 10 years (Fig. 2C.1). There was no difference for death when comparing high CV with low and medium CV (Fig. 2C.2) (Supplemental Table 3).

##### Kidney Donor Profile Index greater or equal than 85%

For low CV and medium CV there was no difference in DCGL or death when comparing with high CV (Supplemental Table 4) (Fig. 2D.1 and D.2).

##### Donors with acute kidney injury

Compared to high CV, there was no evidence that low or medium CV was associated with different risk for DCGL at any time points (Fig. 2E.1). However, we observed an increased risk of death for low CV at 3 month (aHR = 1.48; 95% CI, 1.04–2.10), 5 year (aHR = 1.13; 95% CI, 1.00–1.28) but not at 1 year or 10 years when compared with high CV (Fig. 2E.2) (Supplemental Table 5).

##### Donors with Hepatitis C

For low and medium CV, no statistically significant difference in DCGL or death was observed, when compared with high CV. However, medium CV was associated with lower risk of death at 10 years when compared with high CV (aHR = 0.87; 95% CI, 0.76–0.99), but not statistically significant different at 3 months, 1 year, or 5 years (Supplemental Table 6) (Fig. 2F.1 and F.2).

#### Kaplan–meier analysis

**Fig. 3:**
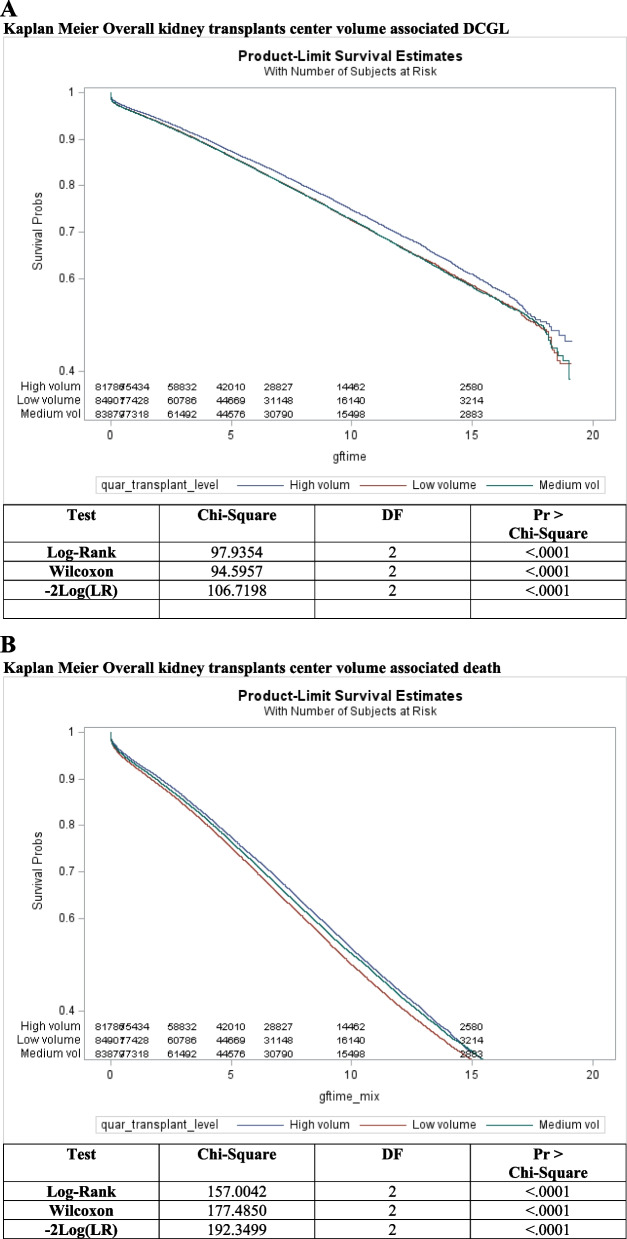
**A** Kaplan Meier Overall kidney transplants center volume associated DCGL. **B** Kaplan Meier Overall kidney transplants center volume associated death

A statistically significant difference in DCGL by center volume was observed (*p* < 0.001) and overall death (*p* < 0.001) (Fig. 3A and B).

## Discussion

Accepting kidneys from high-risk donors and performing kidney transplantation in high-risk recipients requires adequate staff support to monitor their post-transplant outcomes. In this large, national cohort analysis, when examining high-risk recipient subgroups, we found that low CV (compared to high CV) was associated with higher risk of death in elderly (age ≥ 70 years) and higher risk of graft failure in obese patients (BMI ≥ 35 kg/m^2^) at short- and long-term follow-up.

Elderly recipients require more intense medical care and closer follow up after transplantation. The number of elderly recipients continue to increase over the last 10 years [8]. A recent report showed an increase of elderly recipients from 17.6% in 2009 to 24.2% in 2019 [22]. Older recipients are associated with an increased number of comorbidities and at higher risk of infection, cardiovascular diseases, and malignancies, and they are more prevalent to be frail [9, 23–28]. In terms of obesity, it has been associated with increased risk for peritransplant complications, including delayed graft function, wound infections, and graft loss [29–32]. Resources and care practices to manage these complications might be better at high CV, resulting in better outcomes as reported in our paper.

The cause of higher risk of DCGL in obese recipients and death in elderly recipients in low volume centers is most likely multifactorial. Higher volume centers presumably have a complex multidisciplinary team, and broader resources for management and follow up [33, 34]. Higher volume centers might have a larger number of transplant nephrologists and surgeons with different expertise and interests. The impact of a large specialized network of transplant coordinators likely also helps manage and follow up patients with tailored protocols that are needed to improve patient and graft survival. In addition, high volume centers are more likely to have increased availability of other advanced specialties such as transplant cardiology, transplant infectious disease and oncology that may influence outcomes. High Volume Center for each specific high -risk group for elderly and obese might have lower threshold to accept these population that they have practice care specific for them and prepare for potential for the complications.

When compared with high center volume, our study did not find any difference of DCGL or death for low center volume in patients receiving kidneys from donors with AKI, HCV + , or high KDPI. Given the increasing prevalence of KT using kidneys from donors with KDPI ≥ 85%, AKI, and HCV + , it is important to evaluate what factors contribute to the improved outcomes [2, 35, 36].

Our findings for high risk donors are consistent with prior publications that selected deceased kidney donors with AKI were not associated with higher risk of graft failure or death compared to those receiving non-AKI kidneys [4, 5, 37–40]. In the new era of effective direct acting antivirals (DAA), patients receiving kidneys from donors with HCV + do not seem to have an increased risk when compared with those receiving kidneys from donors with seronegative hepatitis C [6, 41, 42]. Our study did not asses the effect of DAA at long term as this were started in 2014 and our study population included patients transplanted up to 2016. High KDPI is a well-known risk factor to have decreased survival of the allograft [43]. Our study showed center volume by KDPI did not seem to play a role in DCGL and death.

Our study has several important strengths. To our knowledge, this is one of the first studies to highlight the outcome implications of KT CV for specific risk factors in the current era with the increasing prevalence of having higher risk donors with high KDPI, donor with AKI and donor with HCV + . Second, we used national data that allowed us to include a large number of kidney transplants with long-term follow-up for several time points of interest. There are alsolimitations. First, it is a retrospective study based on registry data, which is limited by available variables and existing data quality. Second, the analyses did not account for patient socioeconomic status, which might impact transplant outcomes.

In conclusion, elderly patients who received KT in low-volume centers had increased risk of death compared to those who received KT in high volume centers. Moderate obese recipients who received KT in low-volume centers had increased risk of graft loss compared to those who received KT in high volume centers. Future studies should seek to identify care processes that support optimal outcomes after kidney transplantation irrespective of center volume.

## Supplementary Information

**Additional file 1: Supplemental Table 1.** Overall kidney transplants center volume associated Death Censored Graft Failure and Death. **Supplemental Table 2.** Kidney transplants center volume for recipient age> 70 years associated Death Censored Graft Failure and Death . **Supplemental Table 3.** Kidney transplants center volume for Recipient BMI> 35 Kg/m2 associated Death Censored Graft Failure and Death. **Supplemental Table 4.** Kidney transplants center volume for Donors with KDPI>85% associated Death Censored Graft Failure and Death. **Supplemental Table 5.** Kidney transplant center volume for Transplants with Donor AKI with Serum Creatinine > 2 mg/dl associated Death Censored Graft Failure and Death. S**upplemental Table 6.** Kidney transplant center volume for Transplants with Donors with Hepatitis C associated Death Censored Graft Failure and Death.

## Data Availability

The datasets used and/or analysed during the current study are not publicly available due this information from UNOS/OPTN but are available from the corresponding author on reasonable request.

## Abbreviations

DCGL: Death Censored Graft Loss
ESRD: End Stage Renal Disease
eGFR: Estimated glomerular filtration rate
KT: Kidney Transplant
BMI: Body Mass Index
KDPI: Kidney Donor Profile Index
AKI: Acute Kidney Injury

## References

[1] Axelrod DA, Schnitzler MA, Xiao H (2018). An economic assessment of contemporary kidney transplant practice. Am J Transplant.

[2] Collins AJ, Foley RN, Chavers B, et al. 'United States Renal Data System 2011 Annual Data Report: Atlas of chronic kidney disease & end-stage renal disease in the United States. Am J Kidney Dis. 2012;59(1 Suppl 1):A7, e1–420. 10.1053/j.ajkd.2011.11.015 22177944

[3] Port FK, Merion RM, Goodrich NP, Wolfe RA (2006). Recent trends and results for organ donation and transplantation in the United States, 2005. Am J Transplant Off J Am Soc Transplant Am Soc Transplant Surg.

[4] Heilman RL, Smith ML, Kurian SM (2015). Transplanting Kidneys from Deceased Donors With Severe Acute Kidney Injury. Am J Transplant Off J Am Soc Transplant Am Soc Transplant Surg.

[5] Heilman RL, Smith ML, Smith BH (2019). Long-term Outcomes Following Kidney Transplantation From Donors With Acute Kidney Injury. Transplantation.

[6] Potluri VS, Goldberg DS, Mohan S (2019). National Trends in Utilization and 1-Year Outcomes with Transplantation of HCV-Viremic Kidneys. J Am Soc Nephrol.

[7] Dahmen M, Becker F, Pavenstädt H, Suwelack B, Schütte-Nütgen K, Reuter S (2019). Validation of the Kidney Donor Profile Index (KDPI) to assess a deceased donor’s kidneys’ outcome in a European cohort. Sci Rep.

[8] Lentine KL, Pastan S, Mohan S (2021). A roadmap for innovation to advance transplant access and outcomes: a position statement from the national kidney foundation. Am J Kidney Dis.

[9] Knoll GA (2013). Kidney transplantation in the older adult. Am J Kidney Dis.

[10] Gaston RS, Fieberg A, Helgeson ES (2020). Late Graft Loss After Kidney Transplantation: Is "Death With Function" Really Death With a Functioning Allograft?. Transplantation.

[11] Kwan JM, Hajjiri Z, Metwally A, Finn PW, Perkins DL (2016). Effect of the Obesity Epidemic on Kidney Transplantation: Obesity Is Independent of Diabetes as a Risk Factor for Adverse Renal Transplant Outcomes. PLoS ONE.

[12] Aziz F, Ramadorai A, Parajuli S (2020). Obesity: An Independent Predictor of Morbidity and Graft Loss after Kidney Transplantation. Am J Nephrol.

[13] Asrani SK, Kim WR, Edwards EB (2013). Impact of the center on graft failure after liver transplantation. Liver Transplantation.

[14] Arnaoutakis GJ, George TJ, Allen JG (2012). Institutional volume and the effect of recipient risk on short-term mortality after orthotopic heart transplant. J Thorac Cardiovasc Surg.

[15] Kilic A, George TJ, Beaty CA, Merlo CA, Conte JV, Shah AS (2012). The effect of center volume on the incidence of postoperative complications and their impact on survival after lung transplantation. J Thorac Cardiovasc Surg.

[16] Shuhaiber JH, Moore J, Dyke DB (2010). The effect of transplant center volume on survival after heart transplantation: a multicenter study. J Thorac Cardiovasc Surg.

[17] Thabut G, Christie JD, Kremers WK, Fournier M, Halpern SD (2010). Survival differences following lung transplantation among US transplant centers. JAMA.

[18] Weiss ES, Allen JG, Meguid RA (2009). The impact of center volume on survival in lung transplantation: an analysis of more than 10,000 cases. Ann Thorac Surg.

[19] Axelrod DA, Guidinger MK, McCullough KP, Leichtman AB, Punch JD, Merion RM (2004). Association of center volume with outcome after liver and kidney transplantation. Am J Transplant Off J Am Soc Transplant Am Soc Transplant Surg.

[20] Schurman SJ, Stablein DM, Perlman SA, Warady BA (1999). Center volume effects in pediatric renal transplantation. A report of the North American Pediatric Renal Transplant Cooperative Study. Pediatric Nephrology (Berlin, Germany).

[21] Alhamad T, Malone AF, Brennan DC (2017). Transplant Center Volume and the Risk of Pancreas Allograft Failure. Transplantation.

[22] Hart A, Lentine KL, Smith JM, et al. OPTN/SRTR 2019 Annual Data Report: Kidney. 2021;21(S2): 21–137. 10.1111/ajt.16502 33595191

[23] Trouillhet I, Benito N, Cervera C (2005). Influence of age in renal transplant infections: cases and controls study. Transplantation.

[24] Martins PN, Pratschke J, Pascher A (2005). Age and immune response in organ transplantation. Transplantation.

[25] Meier-Kriesche H-U, Ojo AO, Hanson JA, Kaplan B (2001). Exponentially increased risk of infectious death in older renal transplant recipients. Kidney Int.

[26] Farrugia D, Mahboob S, Cheshire J (2014). Malignancy-related mortality following kidney transplantation is common. Kidney Int.

[27] Kappes U, Schanz G, Gerhardt U, Matzkies F, Suwelack B, Hohage H (2001). Influence of age on the prognosis of renal transplant recipients. Am J Nephrol.

[28] Legeai C, Andrianasolo RM, Moranne O (2018). Benefits of kidney transplantation for a national cohort of patients aged 70 years and older starting renal replacement therapy. Am J Transplant Off J Am Soc Transplant Am Soc Transplant Surg.

[29] Lynch RJ, Ranney DN, Shijie C, Lee DS, Samala N, Englesbe MJ (2009). Obesity, surgical site infection, and outcome following renal transplantation. Ann Surg.

[30] Meier-Kriesche HU, Kaplan B (2002). Waiting time on dialysis as the strongest modifiable risk factor for renal transplant outcomes: a paired donor kidney analysis. Transplantation.

[31] Aalten J, Christiaans MH, de Fijter H (2006). The influence of obesity on short- and long-term graft and patient survival after renal transplantation. Transplant International.

[32] Molnar MZ, Kovesdy CP, Mucsi I (2011). Higher recipient body mass index is associated with post-transplant delayed kidney graft function. Kidney Int.

[33] Finks JF, Osborne NH, Birkmeyer JD (2011). Trends in hospital volume and operative mortality for high-risk surgery. N Engl J Med.

[34] Birkmeyer JD, Stukel TA, Siewers AE, Goodney PP, Wennberg DE, Lucas FL (2003). Surgeon volume and operative mortality in the United States. N Engl J Med.

[35] Moist LM, Fenton S, Kim JS (2014). Canadian Organ Replacement Register (CORR): reflecting the past and embracing the future. Can J Kidney Health Dis.

[36] Snoeijs MG, Schaubel DE, Hene R (2010). Kidneys from donors after cardiac death provide survival benefit. J Am Soc Nephrol.

[37] Liu C, Hall IE, Mansour S, Thiessen Philbrook HR, Jia Y, Parikh CR (2020). Association of Deceased Donor Acute Kidney Injury With Recipient Graft Survival. JAMA Netw Open.

[38] Oppong YD, Farber JL, Chervoneva I, Martinez Cantarin MP (2016). Correlation of acute tubular injury in reperfusion biopsy with renal transplant outcomes. Clin Transplant.

[39] Yu CC, Ho HC, Yu TM (2014). Kidneys from standard-criteria donors with different severities of terminal acute kidney injury. Transpl Proc.

[40] Anil Kumar MS, Khan SM, Jaglan S (2006). Successful transplantation of kidneys from deceased donors with acute renal failure: Three-year results. Transplantation.

[41] Goldberg DS, Abt PL, Blumberg EA (2017). Trial of Transplantation of HCV-Infected Kidneys into Uninfected Recipients. N Engl J Med.

[42] Molnar MZ, Nair S, Cseprekal O (2019). Transplantation of kidneys from hepatitis C–infected donors to hepatitis C–negative recipients: Single center experience. Am J Transplant.

[43] Gandolfini I, Buzio C, Zanelli P (2014). The Kidney Donor Profile Index (KDPI) of marginal donors allocated by standardized pretransplant donor biopsy assessment: distribution and association with graft outcomes. Am J Transplant Off J Am Soc Transplant Am Soc Transplant Surg.

