# Supplementary material for: The association of center volume with transplant outcomes in selected high-risk groups in kidney transplantation
Source: BMC Nephrol. 2023 Mar 20;24:61. doi: 10.1186/s12882-023-03099-0 (PMC10029277; doi:10.1186/s12882-023-03099-0)
Supplement: Supplementary file 1 — Additional file 1: Supplemental Table 1. Overall kidney transplants center volume associated Death Censored Graft Failure and Death. Supplemental Table 2. Kidney transplants center volume for recipient age> 70 years associated Death Censored Graft Failure and Death . Supplemental Table 3. Kidney transplants center volume for Recipient BMI> 35 Kg/m2 associated Death Censored Graft Failure and Death. Supplemental Table 4. Kidney transplants center volume for Donors with KDPI>85% associated Death Censored Graft Failure and Death. Supplemental Table 5. Kidney transplant center volume for Transplants with Donor AKI with Serum Creatinine > 2 mg/dl associated Death Censored Graft Failure and Death. Supplemental Table 6. Kidney transplant center volume for Transplants with Donors with Hepatitis C associated Death Censored Graft Failure and Death. [file 12882_2023_3099_MOESM1_ESM.docx]

Supplementary Data:

The association of center volume with transplant outcomes in selected high-risk groups in kidney transplantation

Authors: Massini Merzkani, MD, MS^1^ (ORCID: 0000-0003-3979-3418), Su-Hsin Chang, PhD, SM^2^ (ORCID: 0000-0001-5872-9556), Haris Murad, MD^1^, Krista L. Lentine, MD, PhD^3^ (ORCID: 0000-0002-9423-4849), Munis Mattu, MD^1^, Mei Wang^2^, Vangie Hu^4^, Bolin Wang^4^, Yazen Al-Hosni^2^, Obadah Alzahabi^1^,Omar Alomar^1^, Jason Wellen, MD^5^, Tarek Alhamad MD, MS^1,6†^ (ORCID: 0000-0003-4289-0817)

1. Division of Nephrology, Washington University School of Medicine, St. Louis, MO
2. Division of Public Health Sciences, Department of Surgery, Washington University School of Medicine, St. Louis, MO
3. Center for Abdominal Transplantation, Saint Louis University, St. Louis, MO
4. Brown School of Social Work, Washington University in St. Louis, St. Louis, MO
5. Department of Surgery, Washington University in St. Louis, St. Louis, MO
6. Transplant Epidemiology Research Collaboration (TERC), Institute of Public Health, Washington University School of Medicine, St. Louis, MO

^†^Corresponding author:

Tarek Alhamad, MD, MS

Division of Nephrology, Washington University in St. Louis.

4523 Clayton Ave. CB 8126. St. Louis. MO. 63110.

[talhamad@wustl.edu](mailto:talhamad@wustl.edu)

**Supplemental Table 1. Overall kidney transplants center volume associated Death Censored Graft Failure and Death**

| **Time** | **Center Volume** | **Death-censored graft failure** | | | **Death** | | |
| --- | --- | --- | --- | --- | --- | --- | --- |
|  |  | # of events | HR | 95 % CI | # of events | HR | 95 % CI |
| 3M | High volume | 1,835 | Ref | Ref | 1,025 | Ref | Ref |
|  | Low volume | 2,218 | 1.12 | 1.05-1.19 | 1,461 | 1.29 | 1.18-1.40 |
|  | Med volume | 2,229 | 1.11 | 1.05-1.19 | 1,094 | 0.96 | 0.88-1.05 |
| 1Y | High volume | 3,126 | Ref | Ref | 2,679 | Ref | Ref |
|  | Low volume | 3,568 | 1.05 | 0.99-1.10 | 2,737 | 1.15 | 1.10-1.22 |
|  | Med volume | 3,625 | 1.06 | 1.01-1.11 | 3,372 | 0.95 | 0.90-1.003 |
| 5 Y | High volume | 9,029 | Ref | Ref | 10,049 | Ref | Ref |
|  | Low volume | 10,047 | 1.03 | 0.99-1.06 | 11,646 | 1.07 | 1.04-1.10 |
|  | Med volume | 10,231 | 1.03 | 0.99-1.06 | 10,393 | 0.97 | 0.94-0.99 |
| 10 Y | High volume | 13,415 | Ref | Ref | 17,974 | Ref | Ref |
|  | Low volume | 15,200 | 1.04 | 1.02-1.07 | 21,056 | 1.07 | 1.05-1.09 |
|  | Med volume | 15,211 | 1.03 | 1.003-1.05 | 18,797 | 0.98 | 0.96-1.001 |

Low volume n=84,903; Med volume n=83,883; High volume n= 81,788

**Supplemental Table 2. Kidney transplants center volume for recipient age> 70 years associated Death Censored Graft Failure and Death**

| **Time** | **Center Volume** | **Death-censored graft failure** | | | **Death** | | |
| --- | --- | --- | --- | --- | --- | --- | --- |
|  |  | # of events | HR | 95 % CI | # of events | HR | 95 % CI |
| 3M | High volume | 130 | Ref | Ref | 125 | Ref | Ref |
|  | Low volume | 137 | 1.09 | 0.85-1.40 | 175 | 1.34 | 1.05-1.70 |
|  | Med volume | 131 | 1.02 | 0.79-1.31 | 167 | 1.26 | 0.99-1.61 |
| 1Y | High volume | 251 | Ref | Ref | 361 | Ref | Ref |
|  | Low volume | 241 | 1.02 | 0.85-1.23 | 433 | 1.19 | 1.03-1.38 |
|  | Med volume | 230 | 0.92 | 0.77-1.11 | 426 | 1.16 | 1.005-1.34 |
| 5 Y | High volume | 506 | Ref | Ref | 1,379 | Ref | Ref |
|  | Low volume | 484 | 1.02 | 0.90-1.16 | 1,531 | 1.12 | 1.04-1.20 |
|  | Med volume | 456 | 0.92 | 0.80-1.05 | 1,522 | 1.11 | 1.03-1.19 |
| 10 Y | High volume | 630 | Ref | Ref | 2,339 | Ref | Ref |
|  | Low volume | 606 | 1.01 | 0.90-1.10 | 2,464 | 1.07 | 1.01-1.14 |
|  | Med volume | 574 | 0.91 | 0.81-1.03 | 2,518 | 1.09 | 1.03-1.15 |

Low volume n=5,254; Med volume n=5,192; High volume n= 5,329

**Supplemental Table 3. Kidney transplants center volume for Recipient BMI> 35 Kg/m^2^ associated Death Censored Graft Failure and Death**

| **Time** | **Center Volume** | **Death-censored graft failure** | | | **Death** | | |
| --- | --- | --- | --- | --- | --- | --- | --- |
|  |  | # of events | HR | 95 % CI | # of events | HR | 95 % CI |
| 3M | High volume | 206 | Ref | Ref | 105 | Ref | Ref |
|  | Low volume | 326 | 1.56 | 1.30-1.86 | 176 | 1.50 | 1.17-1.93 |
|  | Med volume | 299 | 1.32 | 1.10-1.59 | 128 | 1.06 | 0.81-1.38 |
| 1Y | High volume | 378 | Ref | Ref | 303 | Ref | Ref |
|  | Low volume | 493 | 1.27 | 1.11-1.46 | 396 | 1.22 | 1.05-1.43 |
|  | Med volume | 448 | 1.08 | 0.94-1.25 | 305 | 0.91 | 0.77-1.07 |
| 5 Y | High volume | 1,068 | Ref | Ref | 1,139 | Ref | Ref |
|  | Low volume | 1,239 | 1.14 | 1.05-1.24 | 1,245 | 1.05 | 0.96-1.14 |
|  | Med volume | 1,242 | 1.06 | 0.98-1.16 | 1,134 | 0.92 | 0.84-1.00 |
| 10 Y | High volume | 1,544 | Ref | Ref | 1,978 | Ref | Ref |
|  | Low volume | 1,769 | 1.11 | 1.03-1.19 | 2,159 | 1.03 | 0.97-1.10 |
|  | Med volume | 1,835 | 1.07 | 1.001-1.15 | 2,083 | 0.97 | 0.91-1.03 |

Low volume n=8,630; Med volume n=8,674; High volume n= 8,672

**Supplemental Table 4. Kidney transplants center volume for Donors with KDPI>85% associated Death Censored Graft Failure and Death**

| **Time** | **Center Volume** | **Death-censored graft failure** | | | **Death** | | |
| --- | --- | --- | --- | --- | --- | --- | --- |
|  |  | # of events | HR | 95 % CI | # of events | HR | 95 % CI |
| 3M | High volume | 310 | Ref | Ref | 171 | Ref | Ref |
|  | Low volume | 322 | 1.04 | 0.88-1.23 | 171 | 0.96 | 0.77-1.20 |
|  | Med volume | 342 | 1.06 | 0.90-1.24 | 203 | 1.10 | 0.89-1.36 |
| 1Y | High volume | 571 | Ref | Ref | 463 | Ref | Ref |
|  | Low volume | 563 | 0.95 | 0.84-1.07 | 454 | 0.91 | 0.80-1.05 |
|  | Med volume | 583 | 0.93 | 0.83-1.05 | 507 | 1.02 | 0.89-1.16 |
| 5 Y | High volume | 1,203 | Ref | Ref | 1,402 | Ref | Ref |
|  | Low volume | 1,222 | 0.98 | 0.90-1.06 | 1,551 | 1.01 | 0.94-1.09 |
|  | Med volume | 1,322 | 0.98 | 0.900-1.060 | 1,594 | 1.04 | 0.96-1.12 |
| 10 Y | High volume (n=5,695) | 1,584 | Ref | Ref | 2,165 | Ref | Ref |
|  | Low volume (n= 5,873) | 1,635 | 0.98 | 0.91-1.06 | 2,518 | 1.05 | 0.99-1.11 |
|  | Med volume (n=5,917) | 1,719 | 0.96 | 0.89-1.03 | 2,485 | 1.04 | 0.98-1.11 |

Low volume n=5,873; Med volume n=5,917; High volume n= 5,695

**Supplemental Table 5. Kidney transplant center volume for Transplants with Donor AKI with Serum Creatinine > 2 mg/dl associated Death Censored Graft Failure and Death**

| **Time** | **Center Volume** | **Death-censored graft failure** | | | **Death** | | |
| --- | --- | --- | --- | --- | --- | --- | --- |
|  |  | # of events | HR | 95 % CI | # of events | HR | 95 % CI |
| 3M | High volume | 129 | Ref | Ref | 62 | Ref | Ref |
|  | Low volume | 134 | 1.15 | 0.89-1.49 | 85 | 1.48 | 1.04-2.10 |
|  | Med volume | 144 | 1.23 | 0.95-1.58 | 61 | 1.12 | 0.77-1.62 |
| 1Y | High volume | 210 | Ref | Ref | 171 | Ref | Ref |
|  | Low volume | 200 | 1.00 | 0.81-1.23 | 187 | 1.15 | 0.92-1.43 |
|  | Med volume | 216 | 1.06 | 0.86-1.30 | 161 | 1.00 | 0.79-1.25 |
| 5 Y | High volume | 505 | Ref | Ref | 571 | Ref | Ref |
|  | Low volume | 536 | 1.06 | 0.93-1.20 | 629 | 1.13 | 1.00-1.28 |
|  | Med volume | 552 | 1.03 | 0.91-1.17 | 568 | 1.00 | 0.88-1.13 |
| 10 Y | High volume (n=4,303) | 685 | Ref | Ref | 905 | Ref | Ref |
|  | Low volume (n= 4,228) | 740 | 1.04 | 0.93-1.16 | 1,024 | 1.08 | 0.98-1.19 |
|  | Med volume (n=4,132) | 764 | 1.03 | 0.92-1.15 | 961 | 1.01 | 0.92-1.11 |

Low volume n=4,228; Med volume n= 4,132; High volume n= 4,303

**Supplemental Table 6. Kidney transplant center volume for Transplants with Donors with Hepatitis C associated Death Censored Graft Failure and Death**

| **Time** | **Center Volume** | **Death-censored graft failure** | | | **Death** | | |
| --- | --- | --- | --- | --- | --- | --- | --- |
|  |  | # of events | HR | 95 % CI | # of events | HR | 95 % CI |
| 3M | High volume | 27 | Ref | Ref | 32 | Ref | Ref |
|  | Low volume | 41 | 1.60 | 0.95-2.69 | 38 | 1.39 | 0.84-2.30 |
|  | Med volume | 44 | 1.71 | 1.03-2.86 | 28 | 1.01 | 0.60-1.72 |
| 1Y | High volume | 66 | Ref | Ref | 95 | Ref | Ref |
|  | Low volume | 70 | 1.13 | 0.79-1.62 | 105 | 1.10 | 0.81-1.47 |
|  | Med volume | 92 | 1.35 | 0.96-1.89 | 80 | 0.87 | 0.64-1.19 |
| 5 Y | High volume | 263 | Ref | Ref | 343 | Ref | Ref |
|  | Low volume | 255 | 1.09 | 0.91-1.31 | 327 | 1.00 | 0.85-1.17 |
|  | Med volume | 269 | 1.08 | 0.90-1.30 | 308 | 0.91 | 0.775-1.07 |
| 10 Y | High volume (n=1,406) | 358 | Ref | Ref | 531 | Ref | Ref |
|  | Low volume (n= 1,424) | 373 | 1.16 | 0.99-1.36 | 520 | 0.99 | 0.87-1.12 |
|  | Med volume (n=1,394) | 369 | 1.08 | 0.92-1.26 | 481 | 0.87 | 0.76-0.99 |

Low volume n=1,424; Med volume n= 1,394; High volume n= 1,406
